# Supplementary material for: Discovery and characterization of a hydroxypyridone-3-carboxamide analog as an antiviral lead against orthopoxviruses
Source: Antimicrob Agents Chemother. 2026 Jun 18;70(8):e01922-25. doi: 10.1128/aac.01922-25 (PMC13286052; doi:10.1128/aac.01922-25)
Supplement: Supplementary file 1 [file aac.01922-25-s0001.pdf]

Supplementary Information

Discovery and Characterization of a Hydroxypyridone-3-carboxamide Analog as an Antiviral Lead against Orthopoxviruses

Anil Pant[1], Ajit Jagtap[2], Jiashu Xie[2], Djamal Brahim Belhaouari[1], Wei Xie[2], Jacob P. Mahoney[2], Lara Dsouza[1], Ziyue Wang[1], Zeinab R. Aboezz[1], Tibor Farkas[1], Zhengqiang Wang[2*], Zhilong Yang[1*]

[1]Department of Veterinary Pathobiology, College of Veterinary Medicine & Biomedical Sciences, Texas A&M University, College Station, TX 77843

[2]Center for Drug Design, College of Pharmacy, University of Minnesota, Minneapolis, MN 55455

*Correspondence to: Zhengqiang Wang, wangx472@umn.edu, Zhilong Yang, zhilongyang@tamu.edu

Scheme 1. Synthesis of HPCAm Analog **ZW2038**.

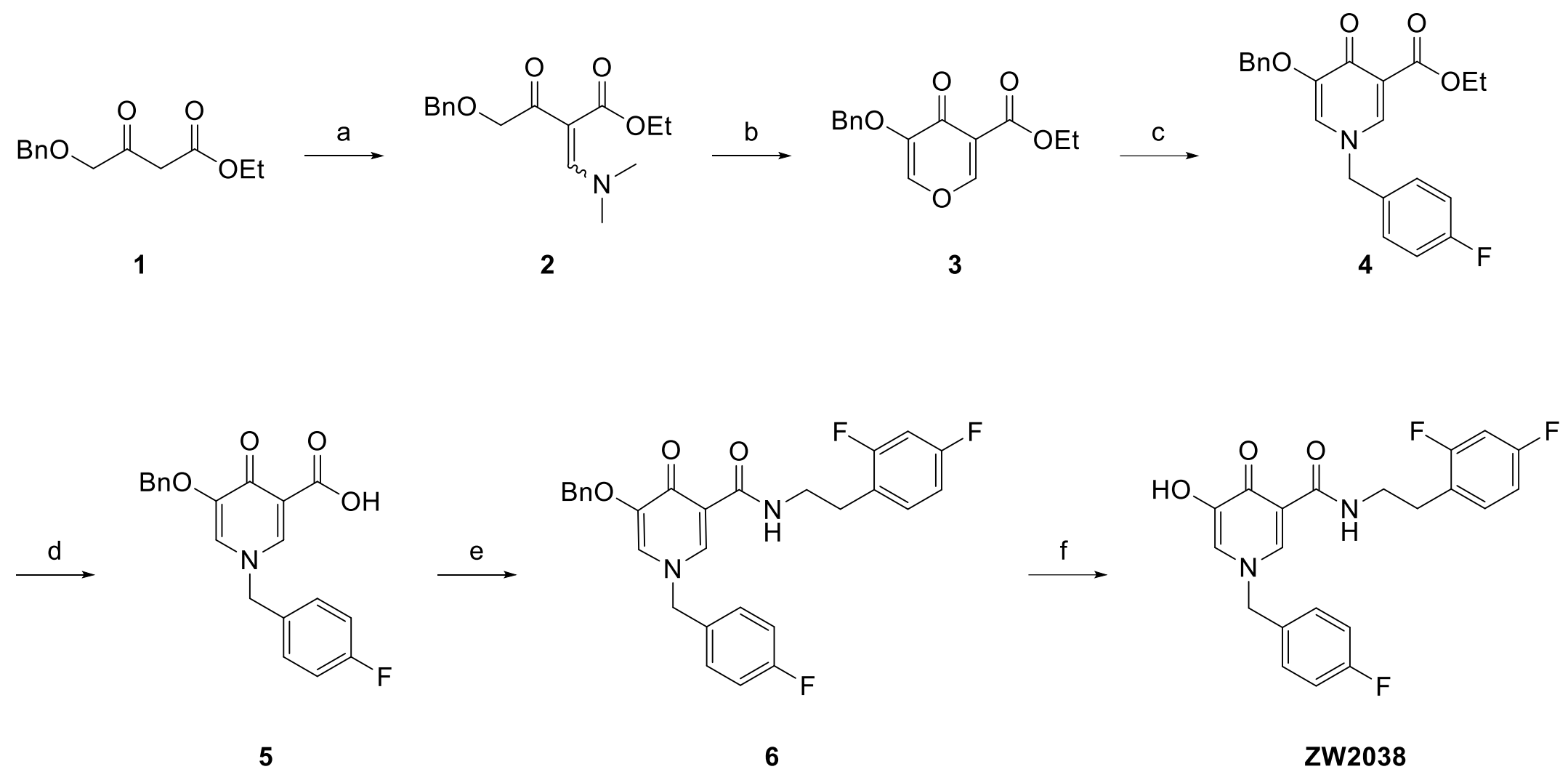

Reagents and conditions: a) DMF-DMA, 100 °C, 78%; b) Ethyl formate, KOtBu, THF, rt, 85%; c) 4F-$PhCH_2CH_2NH_2$, EtOH, microwave: 100 °C, 15 min; d) 2M NaOH, EtOH, 90 °C, 4−6h, 68% (two steps); e) 2,4-di $FPhCH_2NH_2$, HATU, DIPEA, DMF, rt, 12h, 71%; f) TFA, microwave: 100 °C, 10 min, 45%.

*N-(2,4-difluorophenethyl)-1-(4-fluorobenzyl)-5-hydroxy-4-oxo-1,4-dihydropyridine-3-carboxamide (**ZW2038**)*

White solid. ^{1}H NMR (400 MHz, DMSO-*d₆*) δ 10.18 (t, *J* = 5.8 Hz, 1H), 9.14 (s, 1H), 8.48 (d, *J* = 2.3 Hz, 1H), 7.66 (d, *J* = 2.3 Hz, 1H), 7.49 – 7.43 (m, 2H), 7.37 (td, *J* = 8.6, 6.6 Hz, 1H), 7.28 – 7.21 (m, 2H), 7.18 (ddd, *J* = 10.5, 9.4, 2.6 Hz, 1H), 7.02 (tdd, *J* = 8.5, 2.6, 1.0 Hz, 1H), 5.25 (s, 2H), 3.56 (q, *J* = 6.6 Hz, 2H), 2.84 (t, *J* = 6.9 Hz, 2H). ^{13}C NMR (101 MHz, DMSO-d_6) δ 169.9, 164.6, 162.5 (d, *J* = 245.4 Hz), 161.4 (dd, *J* = 244.9, 12.1 Hz), 161.1 (dd, *J* = 247.4, 12.2 Hz), 149.8, 139.9, 132.9 (d, *J* = 3.0 Hz), 132.5 (dd, *J* = 10.1, 6.5 Hz), 130.8 (d, *J* = 8.1 Hz), 122.8 (dd, *J* = 16.2, 3.0 Hz), 122.6, 116.3 (d, *J* = 22.2 Hz), 115.7, 111.8 (dd, *J* = 21.2, 4.0 Hz), 104.1 (t, *J* = 26.3 Hz), 59.2, 38.9, 28.1. ^{19}F NMR (400 MHz, DMSO-d_6) δ -112.97 (p, *J* = 8.1 Hz), -113.53 (tt, *J* = 9.7, 5.5 Hz), -114.27 (q, *J* = 8.8 Hz). HRMS (ESI+): *m/z* calcd for $C_{21}H_{17}F_3N_2O_3$ $[M+H]^+$ 403.1270; found 403.1253.

^{1}H, ^{13}C, ^{19}F-NMR, HPLC and HRMS spectra of **ZW2038**

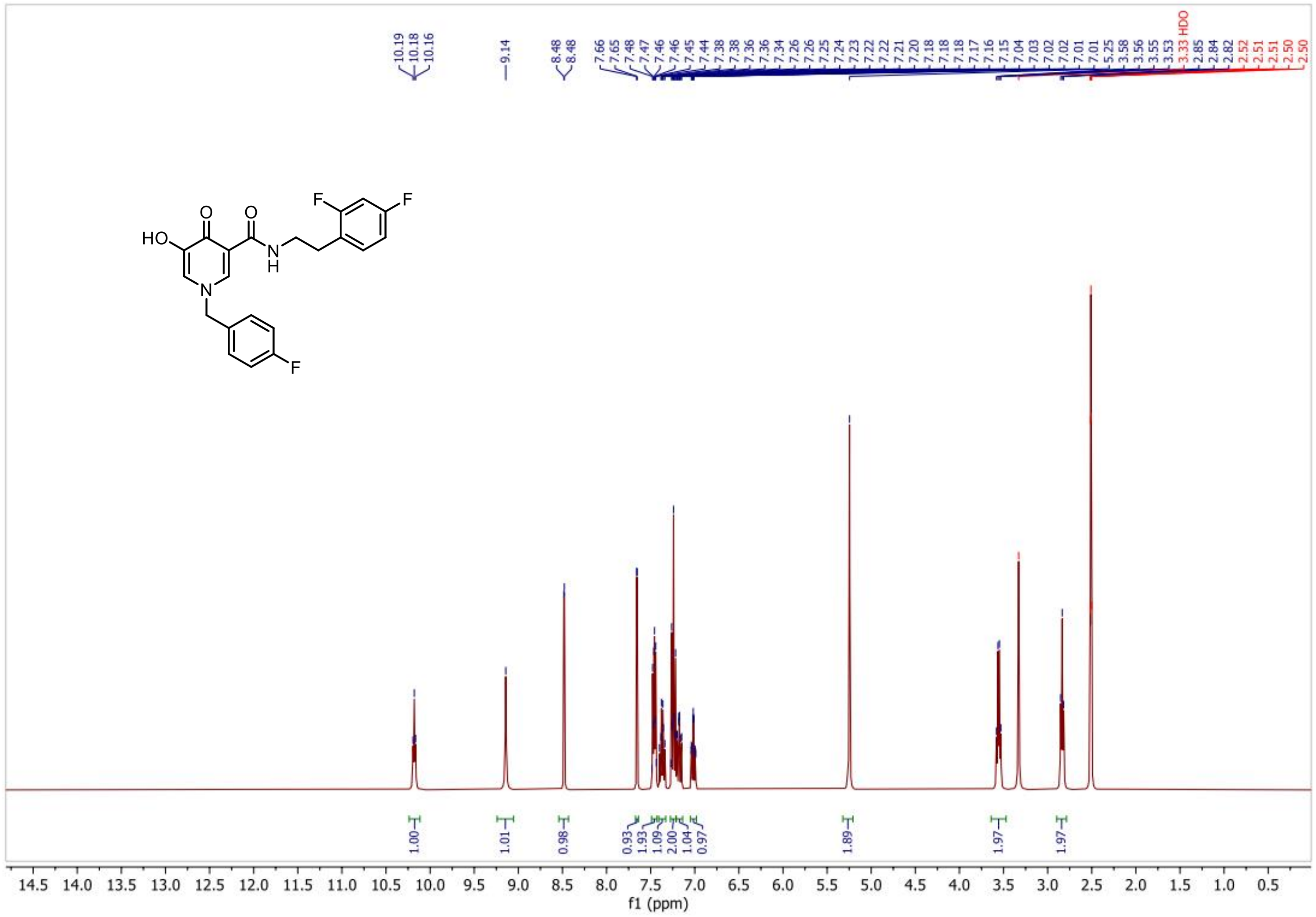

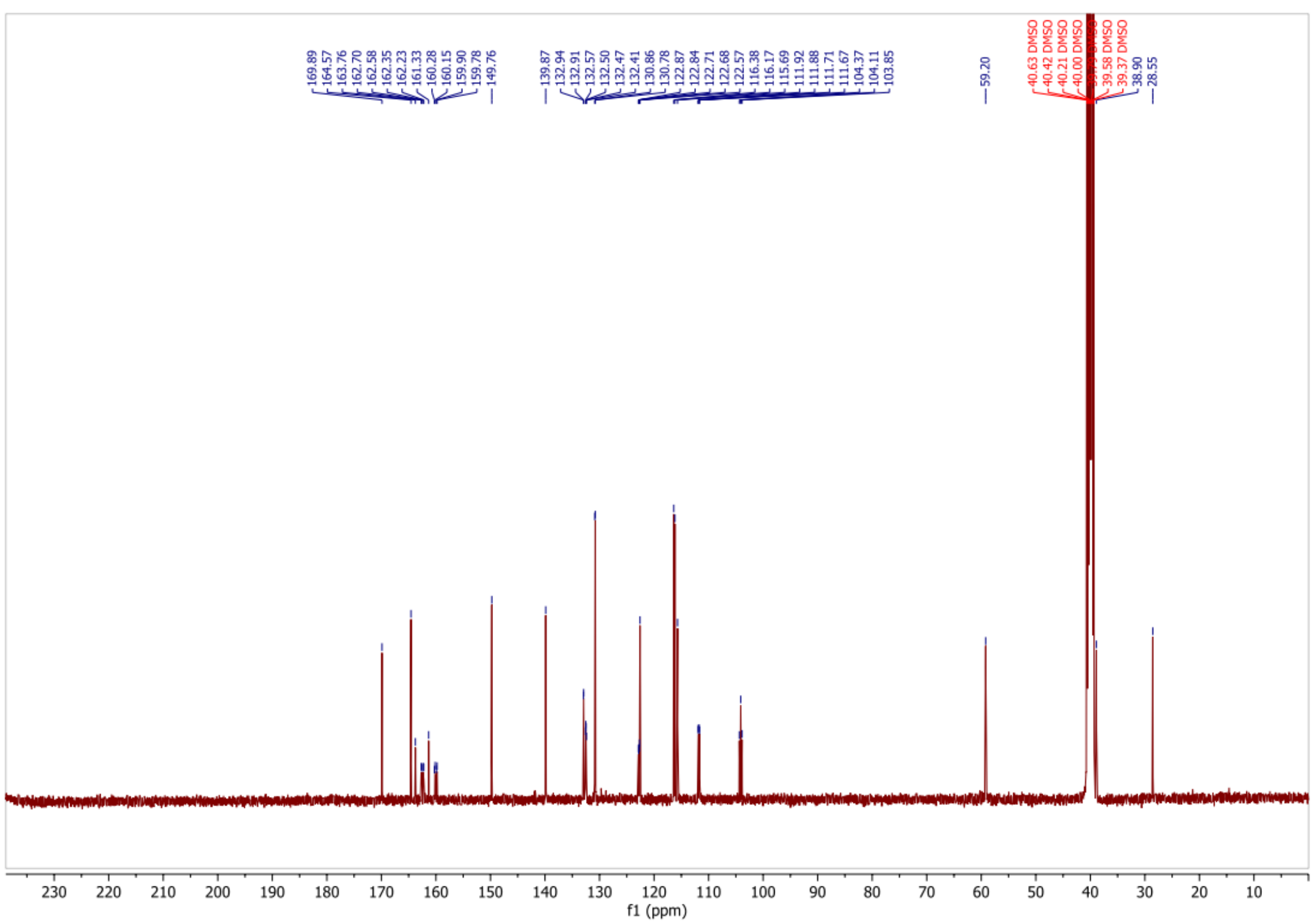

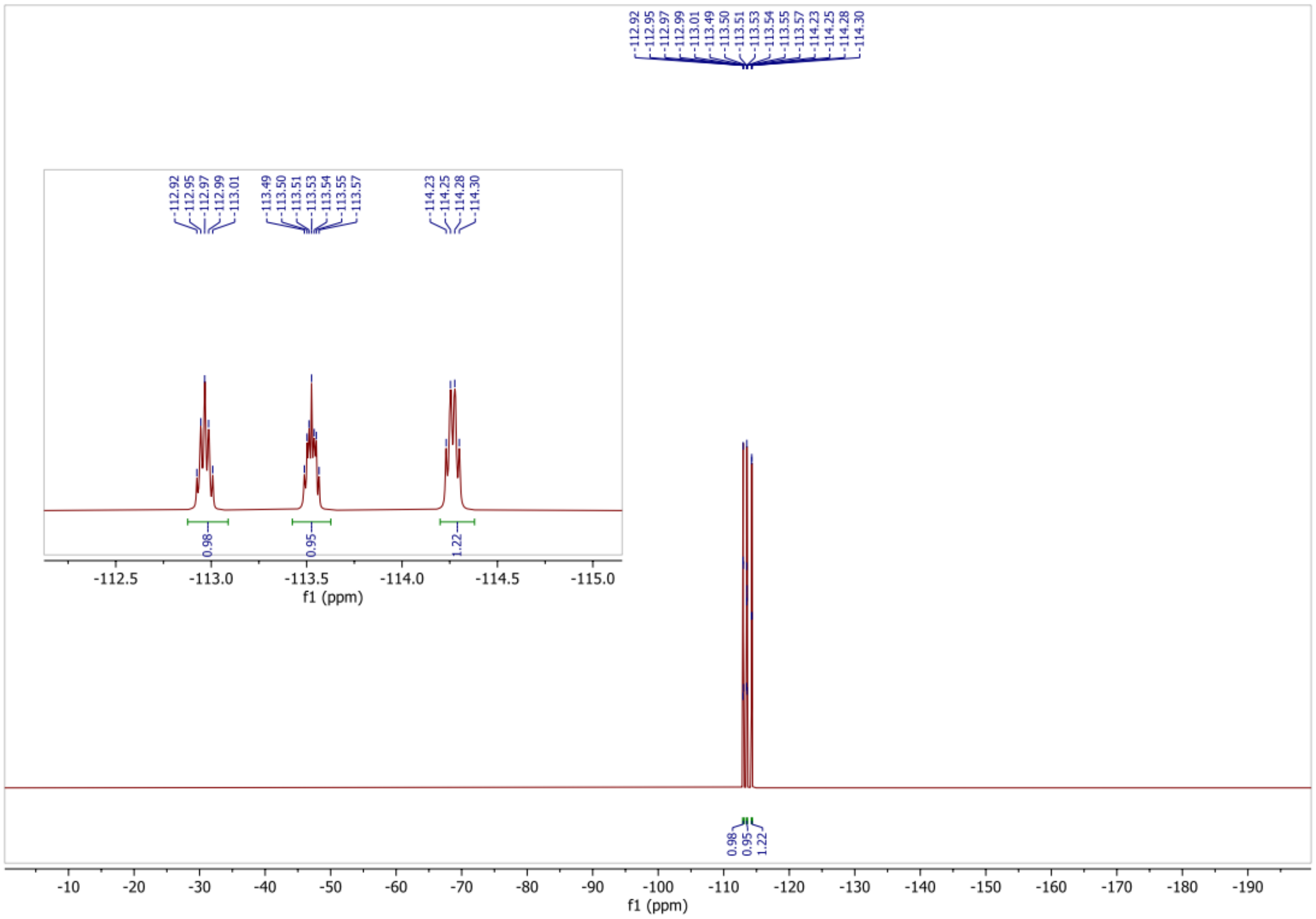

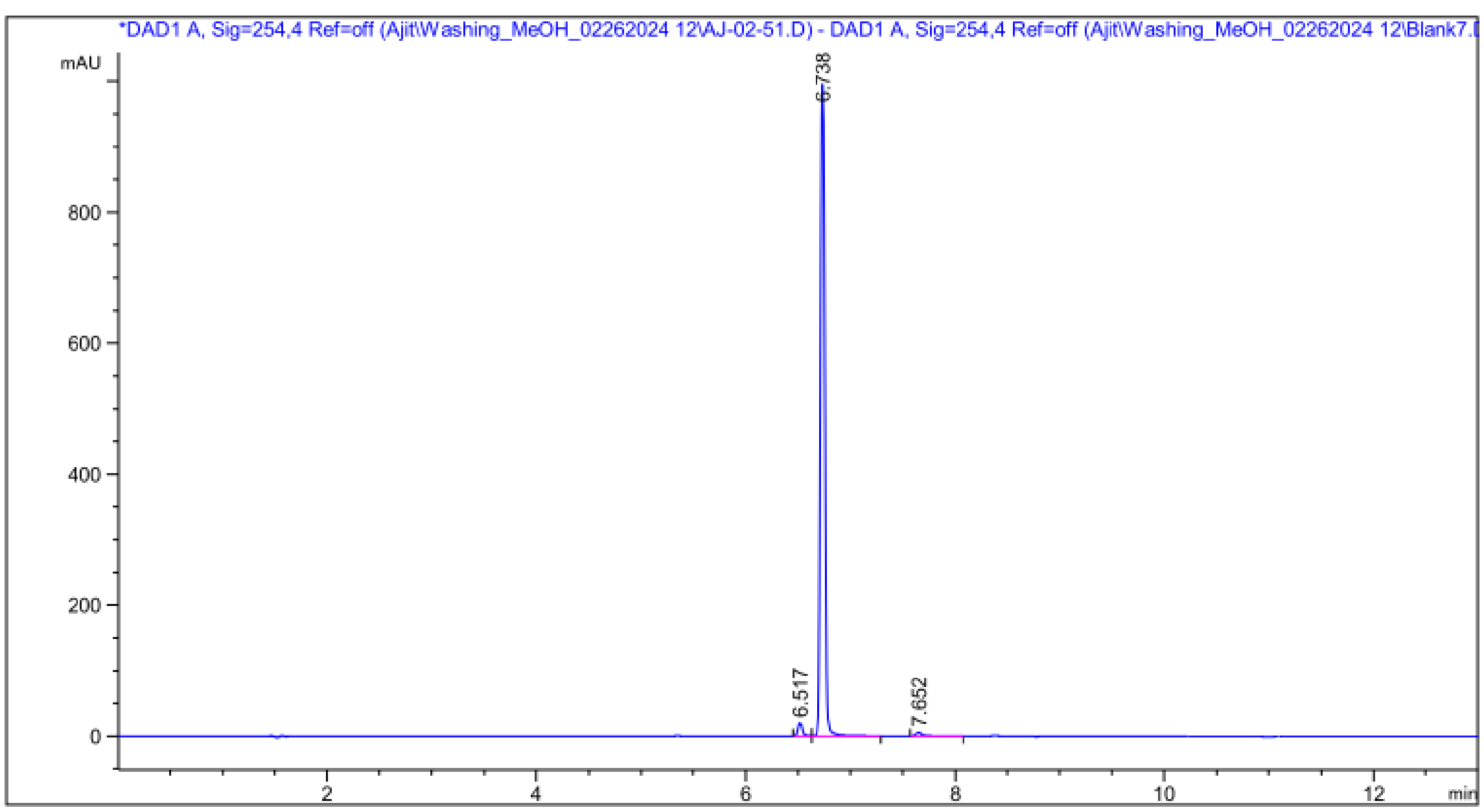

Peak #	RetTime [min]	Type	Width [min]	Area [mAU*s]	Height [mAU]	Area %
1	6.517	BV	0.0489	63.95525	20.23915	2.1619
2	6.738	VB	0.0477	2872.89941	995.06824	97.1130
3	7.652	BB	0.0608	21.45243	5.34214	0.7252
Totals :				2958.30709	1020.64953	

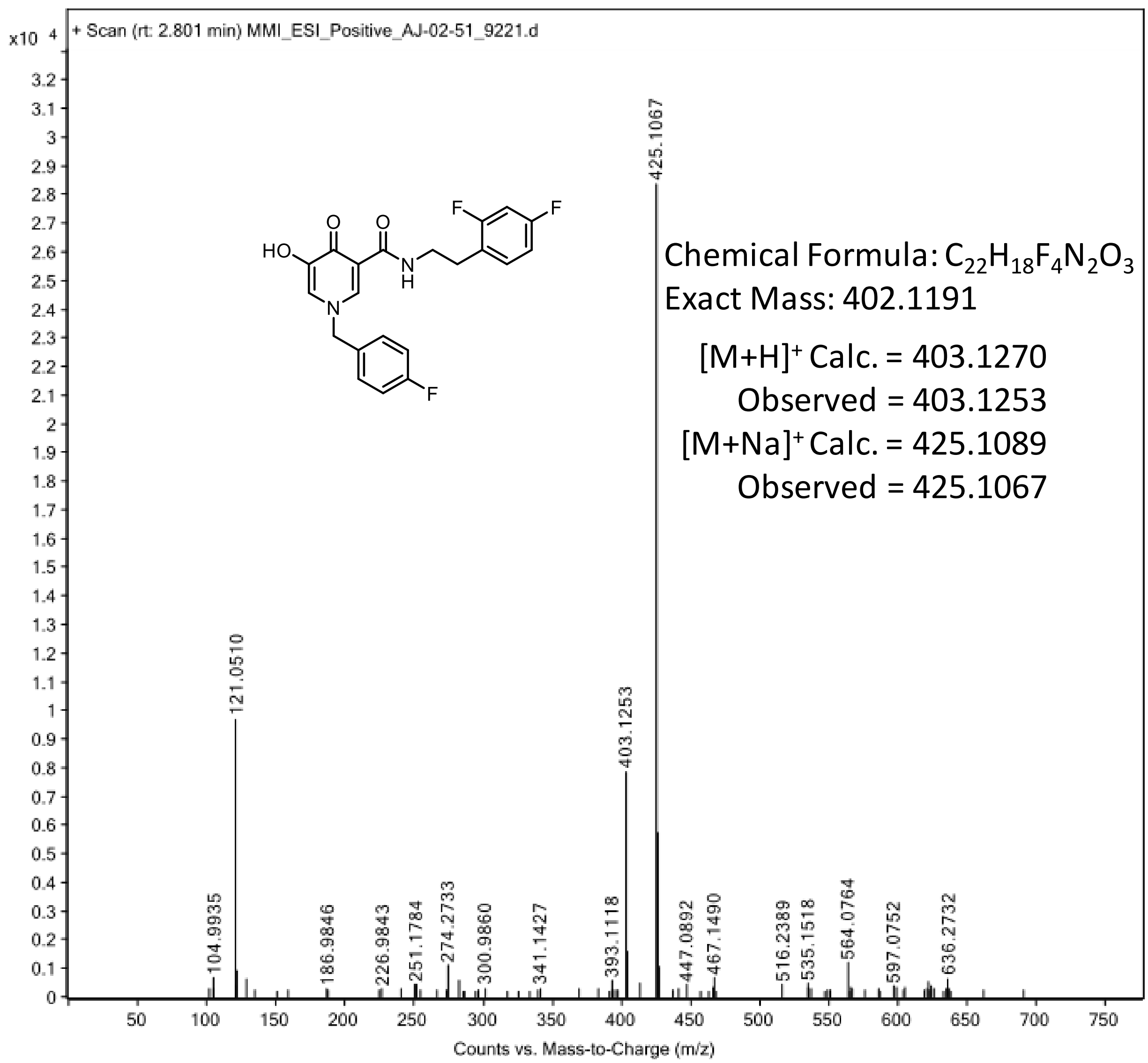
+ Scan (rt: 2.801 min) MMI_ESI_Positive_AJ-02-51_9221.d
x10 4
Chemical Formula: $C_{22}H_{18}F_4N_2O_3$
Exact Mass: 402.1191
$[M+H]^+$ Calc. = 403.1270
Observed = 403.1253
$[M+Na]^+$ Calc. = 425.1089
Observed = 425.1067
425.1067
403.1253
121.0510
104.9935
186.9846
226.9843
251.1784
274.2733
300.9860
341.1427
393.1118
447.0892
467.1490
516.2389
535.1518
564.0764
597.0752
636.2732
Counts vs. Mass-to-Charge (m/z)
